# 1-[3-(Hy­droxy­meth­yl)phen­yl]-3-phenyl­urea

**DOI:** 10.1107/S1600536811028315

**Published:** 2011-07-23

**Authors:** Hyeong Choi, Yong Suk Shim, Byung Hee Han, Sung Kwon Kang, Chang Keun Sung

**Affiliations:** a Department of Chemistry, Chungnam National University, Daejeon 305-764, Republic of Korea; b Department of Food Science and Technology, Chungnam National University, Daejeon 305-764, Republic of Korea

## Abstract

In the title compound, C_14_H_14_N_2_O_2_, the dihedral angle between the benzene rings is 23.6 (1)°. The H atoms of the urea NH groups are positioned *syn* to each other. In the crystal, inter­molecular N—H⋯O and O—H⋯O hydrogen bonds link the mol­ecules into a three-dimensional network.

## Related literature

For general background to melanin, see: Kubo *et al.* (2000 ▶); Claus & Decker (2006 ▶). For the development of tyrosinase inhibitors, see: Khan *et al.* (2006 ▶); Kojima *et al.* (1995 ▶); Cabanes *et al.* (1994 ▶); Casañola-Martin *et al.* (2006 ▶); Son *et al.* (2000 ▶); Hong *et al.* (2008 ▶); Lee *et al.* (2007 ▶); Yi *et al.* (2010 ▶); Choi *et al.* (2010 ▶).
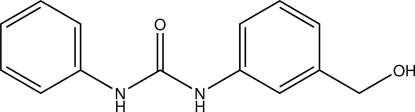


## Experimental

### 

#### Crystal data

C_14_H_14_N_2_O_2_
*M* *_r_* = 242.27
Monoclinic, 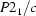

*a* = 14.6207 (8) Å
*b* = 7.0692 (4) Å
*c* = 12.4019 (5) Å
β = 109.818 (3)°
*V* = 1205.90 (11) Å^3^
*Z* = 4
Mo *K*α radiation
μ = 0.09 mm^−1^
*T* = 296 K
0.22 × 0.21 × 0.05 mm

#### Data collection

Bruker SMART CCD area-detector diffractometer
9960 measured reflections
2694 independent reflections
1664 reflections with *I* > 2σ(*I*)
*R*_int_ = 0.052

#### Refinement

*R*[*F* ^2^ > 2σ(*F* ^2^)] = 0.041
*wR*(*F* ^2^) = 0.111
*S* = 0.93
2694 reflections
175 parameters
H atoms treated by a mixture of independent and constrained refinement
Δρ_max_ = 0.14 e Å^−3^
Δρ_min_ = −0.21 e Å^−3^

### 

Data collection: *SMART* (Bruker, 2002 ▶); cell refinement: *SAINT* (Bruker, 2002 ▶); data reduction: *SAINT*; program(s) used to solve structure: *SHELXS97* (Sheldrick, 2008 ▶); program(s) used to refine structure: *SHELXL97* (Sheldrick, 2008 ▶); molecular graphics: *ORTEP-3 for Windows* (Farrugia, 1997 ▶); software used to prepare material for publication: *WinGX* (Farrugia, 1999 ▶).

## Supplementary Material

Crystal structure: contains datablock(s) global, I. DOI: 10.1107/S1600536811028315/im2305sup1.cif

Structure factors: contains datablock(s) I. DOI: 10.1107/S1600536811028315/im2305Isup2.hkl

Supplementary material file. DOI: 10.1107/S1600536811028315/im2305Isup3.cml

Additional supplementary materials:  crystallographic information; 3D view; checkCIF report

## Figures and Tables

**Table 1:** Hydrogen-bond geometry (Å, °)

*D*—H⋯*A*	*D*—H	H⋯*A*	*D*⋯*A*	*D*—H⋯*A*
N7—H7⋯O18^i^	0.87 (2)	2.12 (2)	2.958 (2)	163 (1)
N10—H10⋯O18^i^	0.90 (2)	2.18 (2)	3.031 (2)	157 (1)
O18—H18⋯O9^ii^	0.86 (2)	1.91 (2)	2.763 (2)	175 (2)

Symmetry codes: (i) 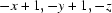; (ii) 
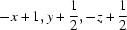
.

**Fig. 1.**
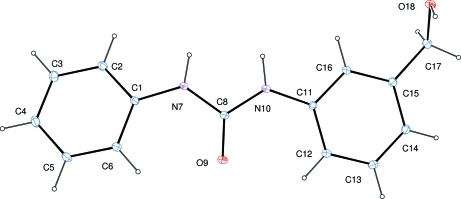
Molecular structure of (I), showing the atom-numbering scheme and 30% probability ellipsoids.

**Fig. 2.**
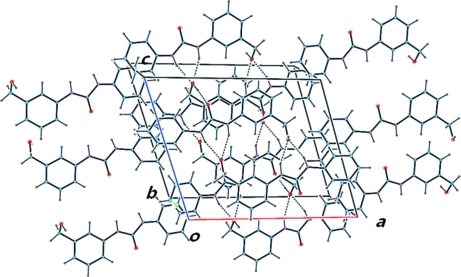
Part of the crystal structure of (I), showing 3-D network of molecules linked by intermolecular N—H···O and O—H···O hydrogen bonds (dashed lines).

## supplementary crystallographic information

### Comment

Melanin plays a major role in human skin protection as well as in undesirable browning of fruits and vegetables (Kubo *et al.*, 2000). Tyrosinase is a key enzyme involved in the first stage of melanin synthesis. Tyrosinase catalyses two distinct reactions involving molecular oxygen, the hydroxylation of tyrosine to *L*-DOPA as a monophenolase and the oxidation of *L*-DOPA to dopaquinone as a diphenolase. Dopaquinone is non-enzymatically converted into dopachrome and dihydroxyindols, which induce the production of melanin pigments (Claus & Decker, 2006). Tyrosinase inhibitors have become increasingly important in both agriculture cosmetic industry and medication due to decreasing the excessive accumulation of pigmentation resulting from the enzyme action (Khan *et al.*, 2006). Numerous potential tyrosinase inhibitors have been discovered from natural and synthetic sources, such as ascorbic acid (Kojima *et al.*, 1995), kojic acid (Cabanes *et al.*, 1994), arbutin (Casañola-Martin *et al.*, 2006) and tropolone (Son *et al.*, 2000). They contain aromatic, methoxy, hydroxyl (Hong *et al.*, 2008; Lee *et al.*, 2007), aldehyde (Yi *et al.*, 2010), and amide (Choi *et al.*, 2010) groups in their structure. Nevertheless, some of their individual activities are either not potent enough to be considered of practical use or not compatible with safety regulations for food and cosmetic additives. Therefore, it is still necessary to search and develop novel tyrosinase inhibitors with potent activities and lower side effects. In our continuing search for tyrosinase inhibitors, we have synthesized the title compound, (I), from the reaction of 3-aminobenzyl alcohol and phenyl isocyanate under ambient condition. Here, the crystal sturucture of (I) is described (Fig.1).

The 3-hydroxymethylphenyl moiety is essentially planar with a mean deviation of 0.010 Å from the corresponding least-squares plane defined by the eight constituent atoms. The dihedral angle between the benzene rings is 23.6 (1) °. The presence of intermolecular N—H···O and O—H···O hydrogen bonds link the molecules into a three-dimensional network (Fig. 2, Table 1). The H atoms of the NH groups of urea are positioned *syn* to each other.

### Experimental

3-Aminobenzyl alcohol and phenyl isocyanate were purchased from Sigma Chemical Co. Solvents used for organic synthesis were redistilled before use. All other chemicals and solvents were of analytical grade and used without further purification. The title compound was prepared from the reaction of 3-aminobenzyl alcohol (0.2 g, 1.0 mmol) with phenyl isocyanate (0.23 g, 1.2 mmol) in acetonitrile (6 ml) with stirring. The reaction was completed within 30 min at room temperature under 1 atm of N_2_ gas. The solvents were removed under reduced pressure. The solids collected and washed with dichloromethane. Removal of the solvent gave a white solid (73%, m.p. 446 K). Single crystals were obtained from an ethanolic solution by slow evaporation of the solvent at room temperature.

### Refinement

H atoms of NH and OH groups were located in a difference Fourier map and refined freely. Rremaining H atoms were positioned geometrically and refined using a riding model with C—H = 0.93–0.97 Å, and with *U*_iso_(H) = 1.2*U*_eq_ (C) for aromatic and methylene H atoms.

### Crystal data

**Table:**

C_14_H_14_N_2_O_2_	*F*(000) = 512
*M**_r_* = 242.27	*D*_x_ = 1.334 Mg m^−^^3^
Monoclinic, *P*2_1_/*c*	Mo *K*α radiation, λ = 0.71073 Å
Hall symbol: -P 2ybc	Cell parameters from 2624 reflections
*a* = 14.6207 (8) Å	θ = 3.2–28.0°
*b* = 7.0692 (4) Å	µ = 0.09 mm^−^^1^
*c* = 12.4019 (5) Å	*T* = 296 K
β = 109.818 (3)°	Plate, colourless
*V* = 1205.90 (11) Å^3^	0.22 × 0.21 × 0.05 mm
*Z* = 4	

### Data collection

**Table:**

Bruker SMART CCD area-detector diffractometer	*R*_int_ = 0.052
φ and ω scans	θ_max_ = 27.5°, θ_min_ = 3.0°
9960 measured reflections	*h* = −15→18
2694 independent reflections	*k* = −9→6
1664 reflections with *I* > 2σ(*I*)	*l* = −16→9

### Refinement

**Table:**

Refinement on *F*^2^	0 restraints
Least-squares matrix: full	H atoms treated by a mixture of independent and constrained refinement
*R*[*F*^2^ > 2σ(*F*^2^)] = 0.041	*w* = 1/[σ^2^(*F*_o_^2^) + (0.0584*P*)^2^] where *P* = (*F*_o_^2^ + 2*F*_c_^2^)/3
*wR*(*F*^2^) = 0.111	(Δ/σ)_max_ < 0.001
*S* = 0.93	Δρ_max_ = 0.14 e Å^−^^3^
2694 reflections	Δρ_min_ = −0.21 e Å^−^^3^
175 parameters	

### Special details

**Table:**

Geometry. All e.s.d.'s (except the e.s.d. in the dihedral angle between two l.s. planes) are estimated using the full covariance matrix. The cell e.s.d.'s are taken into account individually in the estimation of e.s.d.'s in distances, angles and torsion angles; correlations between e.s.d.'s in cell parameters are only used when they are defined by crystal symmetry. An approximate (isotropic) treatment of cell e.s.d.'s is used for estimating e.s.d.'s involving l.s. planes.

### Fractional atomic coordinates and isotropic or equivalent isotropic displacement parameters (Å^2^)

**Table:**

	*x*	*y*	*z*	*U*_iso_*/*U*_eq_	
C1	0.13667 (11)	0.36148 (19)	0.11590 (11)	0.0385 (3)	
C2	0.07277 (11)	0.2820 (2)	0.01677 (13)	0.0471 (4)	
H2	0.0933	0.2595	−0.0453	0.057*	
C3	−0.02094 (12)	0.2359 (2)	0.00922 (14)	0.0546 (4)	
H3	−0.0632	0.1839	−0.0581	0.066*	
C4	−0.05198 (12)	0.2664 (2)	0.10023 (15)	0.0581 (5)	
H4	−0.1149	0.2343	0.0955	0.07*	
C5	0.01144 (13)	0.3456 (3)	0.19916 (15)	0.0619 (5)	
H5	−0.0093	0.3672	0.2611	0.074*	
C6	0.10517 (12)	0.3932 (2)	0.20757 (12)	0.0524 (4)	
H6	0.147	0.4464	0.2748	0.063*	
N7	0.22912 (9)	0.41144 (18)	0.11382 (11)	0.0442 (3)	
H7	0.2359 (11)	0.410 (2)	0.0471 (14)	0.049 (4)*	
C8	0.31390 (11)	0.42001 (18)	0.20514 (11)	0.0381 (3)	
O9	0.31723 (8)	0.41011 (14)	0.30535 (8)	0.0495 (3)	
N10	0.39322 (9)	0.44032 (17)	0.17245 (10)	0.0436 (3)	
H10	0.3815 (11)	0.444 (2)	0.0964 (15)	0.057 (5)*	
C11	0.49231 (11)	0.43970 (18)	0.24098 (11)	0.0369 (3)	
C12	0.52658 (11)	0.44241 (19)	0.35949 (12)	0.0454 (4)	
H12	0.4834	0.4428	0.3999	0.055*	
C13	0.62598 (12)	0.4446 (2)	0.41732 (13)	0.0516 (4)	
H13	0.6491	0.4462	0.497	0.062*	
C14	0.69114 (12)	0.4444 (2)	0.35936 (13)	0.0481 (4)	
H14	0.7576	0.4466	0.3997	0.058*	
C15	0.65760 (11)	0.44079 (19)	0.24059 (12)	0.0426 (4)	
C16	0.55875 (11)	0.43755 (19)	0.18285 (12)	0.0423 (4)	
H16	0.5359	0.4338	0.1032	0.051*	
C17	0.72705 (12)	0.4432 (2)	0.17456 (14)	0.0554 (4)	
H17A	0.7921	0.4684	0.2272	0.066*	
H17B	0.7276	0.319	0.1415	0.066*	
O18	0.70325 (8)	0.58030 (19)	0.08550 (9)	0.0559 (3)	
H18	0.6949 (15)	0.685 (3)	0.1155 (18)	0.094 (8)*	

### Atomic displacement parameters (Å^2^)

**Table:**

	*U*^11^	*U*^22^	*U*^33^	*U*^12^	*U*^13^	*U*^23^
C1	0.0402 (9)	0.0407 (8)	0.0384 (7)	0.0035 (6)	0.0184 (6)	0.0040 (6)
C2	0.0471 (10)	0.0508 (9)	0.0476 (8)	0.0011 (7)	0.0216 (7)	−0.0047 (7)
C3	0.0484 (11)	0.0514 (10)	0.0634 (10)	−0.0044 (8)	0.0181 (8)	−0.0058 (8)
C4	0.0434 (11)	0.0665 (11)	0.0697 (11)	0.0000 (8)	0.0260 (9)	0.0141 (9)
C5	0.0524 (12)	0.0909 (13)	0.0525 (10)	0.0108 (10)	0.0309 (9)	0.0147 (9)
C6	0.0459 (11)	0.0747 (11)	0.0400 (8)	0.0060 (8)	0.0190 (7)	0.0029 (8)
N7	0.0395 (8)	0.0641 (9)	0.0327 (6)	−0.0023 (6)	0.0169 (6)	0.0007 (6)
C8	0.0433 (9)	0.0383 (8)	0.0361 (7)	−0.0014 (6)	0.0176 (6)	−0.0006 (6)
O9	0.0509 (7)	0.0673 (7)	0.0339 (5)	−0.0055 (5)	0.0193 (5)	−0.0011 (5)
N10	0.0411 (8)	0.0609 (8)	0.0315 (6)	−0.0035 (6)	0.0159 (5)	0.0003 (6)
C11	0.0389 (9)	0.0362 (8)	0.0353 (7)	−0.0021 (6)	0.0121 (6)	−0.0006 (6)
C12	0.0494 (11)	0.0538 (9)	0.0361 (7)	−0.0015 (7)	0.0185 (7)	−0.0032 (7)
C13	0.0521 (11)	0.0638 (11)	0.0354 (7)	0.0000 (8)	0.0103 (7)	−0.0011 (7)
C14	0.0424 (10)	0.0532 (10)	0.0452 (8)	0.0018 (7)	0.0103 (7)	−0.0009 (7)
C15	0.0436 (10)	0.0410 (8)	0.0455 (8)	0.0011 (7)	0.0181 (7)	−0.0005 (6)
C16	0.0421 (10)	0.0507 (9)	0.0354 (7)	−0.0029 (7)	0.0148 (6)	−0.0011 (6)
C17	0.0457 (11)	0.0697 (11)	0.0539 (9)	0.0013 (8)	0.0210 (8)	−0.0028 (8)
O18	0.0630 (8)	0.0707 (9)	0.0428 (6)	−0.0048 (6)	0.0294 (6)	−0.0059 (6)

### Geometric parameters (Å, °)

**Table:**

C1—C6	1.3820 (19)	N10—H10	0.900 (17)
C1—C2	1.386 (2)	C11—C12	1.3829 (19)
C1—N7	1.4058 (19)	C11—C16	1.392 (2)
C2—C3	1.380 (2)	C12—C13	1.386 (2)
C2—H2	0.93	C12—H12	0.93
C3—C4	1.369 (2)	C13—C14	1.374 (2)
C3—H3	0.93	C13—H13	0.93
C4—C5	1.381 (2)	C14—C15	1.386 (2)
C4—H4	0.93	C14—H14	0.93
C5—C6	1.380 (2)	C15—C16	1.379 (2)
C5—H5	0.93	C15—C17	1.505 (2)
C6—H6	0.93	C16—H16	0.93
N7—C8	1.3678 (19)	C17—O18	1.421 (2)
N7—H7	0.866 (17)	C17—H17A	0.97
C8—O9	1.2291 (15)	C17—H17B	0.97
C8—N10	1.3597 (18)	O18—H18	0.86 (2)
N10—C11	1.4094 (18)		
			
C6—C1—C2	118.88 (14)	C11—N10—H10	115.1 (10)
C6—C1—N7	124.41 (14)	C12—C11—C16	119.05 (14)
C2—C1—N7	116.66 (12)	C12—C11—N10	124.67 (13)
C3—C2—C1	120.73 (14)	C16—C11—N10	116.28 (12)
C3—C2—H2	119.6	C11—C12—C13	119.26 (14)
C1—C2—H2	119.6	C11—C12—H12	120.4
C4—C3—C2	120.39 (16)	C13—C12—H12	120.4
C4—C3—H3	119.8	C14—C13—C12	121.38 (14)
C2—C3—H3	119.8	C14—C13—H13	119.3
C3—C4—C5	119.08 (15)	C12—C13—H13	119.3
C3—C4—H4	120.5	C13—C14—C15	119.84 (15)
C5—C4—H4	120.5	C13—C14—H14	120.1
C6—C5—C4	121.08 (15)	C15—C14—H14	120.1
C6—C5—H5	119.5	C16—C15—C14	118.90 (14)
C4—C5—H5	119.5	C16—C15—C17	119.96 (13)
C5—C6—C1	119.84 (15)	C14—C15—C17	121.13 (14)
C5—C6—H6	120.1	C15—C16—C11	121.56 (13)
C1—C6—H6	120.1	C15—C16—H16	119.2
C8—N7—C1	127.08 (12)	C11—C16—H16	119.2
C8—N7—H7	115.2 (10)	O18—C17—C15	113.44 (13)
C1—N7—H7	116.0 (10)	O18—C17—H17A	108.9
O9—C8—N10	124.20 (14)	C15—C17—H17A	108.9
O9—C8—N7	123.29 (13)	O18—C17—H17B	108.9
N10—C8—N7	112.51 (12)	C15—C17—H17B	108.9
C8—N10—C11	128.77 (12)	H17A—C17—H17B	107.7
C8—N10—H10	115.9 (10)	C17—O18—H18	106.6 (14)

### Hydrogen-bond geometry (Å, °)

**Table:**

*D*—H···*A*	*D*—H	H···*A*	*D*···*A*	*D*—H···*A*
N7—H7···O18^i^	0.87 (2)	2.12 (2)	2.958 (2)	163 (1)
N10—H10···O18^i^	0.90 (2)	2.18 (2)	3.031 (2)	157 (1)
O18—H18···O9^ii^	0.86 (2)	1.91 (2)	2.763 (2)	175 (2)

Symmetry codes: (i) −*x*+1, −*y*+1, −*z*; (ii) −*x*+1, *y*+1/2, −*z*+1/2.
